# The Impact of Mental Health Status on Health Consumption of the Elderly in China

**DOI:** 10.3390/ijerph18126622

**Published:** 2021-06-20

**Authors:** Wen Liu, Guosheng Han, Xiangzi Yan, Xuan Zhang, Guangjie Ning, Armigon Ravshanovich Akhmedov, William Cannon Hunter

**Affiliations:** 1 Business School, Shandong University, Weihai 264209, China; liuwen@sdu.edu.cn (W.L.); 202020040@mail.sdu.edu.cn (X.Y.); gjning@sdu.edu.cn (G.N.); 2 Business School, Shandong University, Jinan 250100, China; zhangxuan@sdu.edu.cn; 3 Department of Hotel Management, Graduate School, Kyung Hee University, Seoul 02447, Korea; armigon@khu.ac.kr; 4 Department of Convention Management, College of Hotel and Tourism, Kyung Hee University, Seoul 02447, Korea

**Keywords:** elderly, health consumption, mental health, Tobit model, truncated data regression, principal component analysis, China

## Abstract

Based on the national baseline survey data of the CHARLS2015, the comprehensive evaluation index of depression degree of the elderly in China is calculated using a principal component analysis method. The Tobit model is used to investigate the influence of mental health status with depression degree as a proxy variable on the health consumption of the elderly in China. The results show that the overall effect and the phased effect of depression on the health consumption of the elderly are positive. In other words, high levels of depression lead to a higher probability and amount of health consumption. Research findings show that chronic illness and increased social activity can weaken the effect of depression on the health spending of the elderly. Fully considering the factors affecting the mental health of elderly people is required to improve their level of mental health. Importantly, the quality audit system of health products and the supervision and management system of the health consumption market should be improved to realize the benign operation of the health consumption market.

## 1. Introduction

The burden of national mental health problems, and their associated social, human rights, and economic consequences [1] have been recognized by the World Health Organization (WHO), which, in response, has chartered the Mental Health Action Plan 2013–2020 that recognizes the role of mental health in achieving national health [2]. In China, social reforms have worked to improve living standards, medical security systems, and medical and health consumption. The “great health” concept of caring for health and the prevention of illness has gained traction. In 2000, China became home to the largest aging population in the world. In 2017, China had 148 million people aged 65 and over, accounting for 11.4 percent of the total population [3]. This aging population has raised concerns regarding the importance of health consumption among the elderly. 

Medical consumption is driven by physicians’ examinations and prescribed interventions, including hospitalization. Health consumption, on the other hand, is associated with the purchase of prescription and non-prescription drugs, and vitamins and supplements. It is also associated with the use of traditional Chinese medicine and treatment methods, and the purchase and use of personal health and exercise equipment. Health consumption reflects the subjective sentiments and cognition of the elderly population and its choices and concerns. Health consumption, therefore, is an indication of mental health. Between 1990 and 2013, the number of people suffering from depression and/or anxiety worldwide increased by almost 50 percent, from 416 million to 615 million. Nearly 10 percent of the world’s population is affected, and mental disorders account for 30 percent of the global non-fatal disease burden [2,3]. According to the WHO, in 2017 more than 20 percent of the world’s elderly population, aged 60 and over, suffered from mental or neurological disorders, with depression being one of the most common, and an additional 3.8 percent of the elderly suffered from anxiety. As a developing country with the largest elderly population in the world, the mental health development of China’s elderly population has become the key to shaping the global health of the elderly. Improving economic conditions and the widespread availability of health products have worked to increase the aging population’s health consumption. Due to mental health concerns, such as depression, elderly people have come to regard health care products and supplements as important commodities for a healthy life. As a result, some profit-driven businesses can take advantage of the psychological weakness of the elderly, making them “suffer” or “gladly accept” expensive, inefficient, or even ineffective healthcare products.

To recognize and promote mental health and well-being among the elderly, as explicitly stated in the United Nations’ sustainable development goals for 2015–2030 [3], and China’s National Plan for Active Response to Population Aging, more research is focusing on the connection between mental health status and health consumption. However, there is a gap in the research concerning the difference between “medical” and “health” consumption, rarely focusing on the concept of health. In addition, previous research generally recognizes the family as the unit of analysis rather than the individual, particularly the elderly. Additionally, mental health is rarely included in economic models. 

The purpose of this paper is to identify the role of mental health factors in health consumption among the elderly in China. It is hypothesized that there is a positive link between mental health factors and the health consumption demand of the elderly in China. It is also hypothesized that by establishing this link, policymakers will be better informed in making decisions regarding the regulation of the health consumption market. The following research goals are implemented:

- To calculate the comprehensive evaluation of the degree of depression among the elderly in China, based on the national baseline survey data of the CHARLS2015.
- To employ the Tobit model to investigate the influence of mental health status, with depression degree as a proxy variable, on health consumption.
- To identify the causal link between depression and health consumption.
- To suggest policy and research implications associated with improving mental health and improving the operation of the health consumption market.

## 2. Literature Review

### 2.1. Demographic Factors Influencing Consumer Demand for Medical and Health Care 

The factors affecting medical and health consumption are relatively complex. Factors include wage level, medical service price, age, education level, and other factors [4]. These factors are commonly used to assess consumer demand across discrete periods of time. Previous studies, however, have focused only on wage level, finding positive correlations with consumers’ demands for health capital and health care consumption. The income factor largely explains the changes in per capita medical expenses in developed countries [5]. Other studies have included other influencing factors, including medical insurance and age structure, finding that a considerable part of medical expenditure had not been properly explained. The discrepancy was attributed to innovations in medical technology [6].

A globally aging population has drawn attention to age structure as a factor in medical and health consumption. Newer studies focus on the analysis of data at the national level. A study on the consumption patterns of middle-aged and elderly people in the United States found that medical and health consumption expenditure is the second-largest expenditure item of American citizens over 50 years old [7]. Other studies have come to mixed conclusions, finding that medical and health care as a luxury product is greatly affected by the differences in income levels [8]. Panel data of OECD countries from 1971 to 2004 revealed that the income elasticity of medical and health consumption was less than previous estimates and that medical care and health care were a necessity rather than a luxury [9]. In China, research on medical and health consumption has focused on three aspects. First, on the factors affecting family health consumption expenditure, such as the basic medical insurance system [10], poverty and medical insurance [11], and intergenerational support [12]. Second, on the urban–rural differences in China’s family medical and health consumption expenditure [13], paying particular attention to the inequality of China’s rural citizens’ medical resource allocation and medical consumption expenditure [14]. Third, on the characteristics of citizens’ medical consumption structure.

### 2.2. Psychological Factors Influencing Consumer Demand for Medical and Health Care

Psychological factors, such as emotional sentiments, also affect consumer demand and purchase decisions. In food consumption, mood affects the choices between so-called hedonic or high-calorie foods versus more healthy options [15]. Gender also affects food choices [16]. Mood also affects choices in product diversity and trust in the advertised characteristics of products [17]. Emotions enhance consumers’ pursuit of social interaction as a form of consumption. It has also been found that social interaction can influence consumers’ emotions through the language, actions, and expressions of people around them, and change their evaluation of products and consumption decisions [18].

### 2.3. Research Gaps in Medical and Health Consumption among the Elderly 

There are three main gaps in medical and health consumption research. First, there is a gap in the research that fails to identify the differences between “medical” and “health” consumption. Medical consumption is associated with medical exams, hospitalization, and related treatments, while health consumption involves choices related to certain diet supplements and alternative medicine, such as Chinese “herbs” and therapies. Medical and health consumption also differ in terms of motivation. Medical expenditures largely occur because of illness, while health expenditures are based on choice. Income may also play a significant role in choice in the context of health consumption, as well as other factors, such as attitude toward life, health awareness, and psychological well-being. Health consumption has the dual nature of capital goods and consumer goods [4]. It can work to prolong life, therefore providing an individual with more time to create wealth. It can also work to compromise longevity when the pursuit of wealth takes priority over health. Health can also be regarded as an output product. Through health consumption expenditure, consumers can simultaneously improve their level of utility and improve their level of health, slowing down the depreciation rate of health as a capital product. In Grossman’s medical resource demand model, medical and health consumption demand is regarded as an induced demand for health [4]. Medical consumption should be regarded as a part of the larger condition of health. 

Second, there is a gap in the research regarding the unit of analysis. Previous research focused on the family rather than on the differences between individuals within the family, particularly in terms of age. For elderly people, work is no longer the focus of their lives. Their motivation for health consumption may be more related to factors other than income, such as mental health and social interaction. The medical and health consumption of the elderly mainly revolves around the concept of old-age care and the pension model. Aging individuals are affected by social security [19] and pensions, as well as the traditional ways in which the Chinese extended family cares for its elderly [14]. Research concerning individuals rather than the family unit have been limited to issues regarding macro policy, rather the micro.

Third, mental health is rarely included in economic models. The abstract analysis involved in the development of theoretical models may overlook the actual psychological conditions associated with the individual. The subjectivities associated with situational factors and driving motivations may run contradictory to a general economic model [20]. The influence of personality and environment on economic research should not be ignored, and psychological factors should be fully considered in economic analysis [21]. In light of these three research gaps, this paper explores the health consumption of the elderly population from a micro-policy perspective. In addition, the role of mental health factors is considered. The comprehensive depression index, which measures the mental health status of the elderly, is also included in the model.

## 3. Research Methods 

### 3.1. Research Content

Psychological factors play a decisive role in health consumption choices. Among the aging, these factors may include poor health and depression. Those who suffer with depression tend to implement short-term self-restraint behavior to achieve immediate emotional relief [22]. Long-term depression may motivate such individuals to seek modes of health consumption that work to relieve the negative emotions associated with such a mood trait. The independent purchase and use of health care products can enhance aging individuals’ confidence in their health status, thus improving their emotional state. Health products may also require professional assistance in order to make certain purchase decisions. Health and fitness equipment often attracts customers to in-store experiences, where the purchase process can offer opportunities for elderly consumers to communicate with others, including sales assistants or special customer groups, relieving the distress of depression. In this study, it is hypothesized that there is a positive link between mental health factors and the health consumption demand of the elderly in China. It is also hypothesized that long-term depression will increase the elderly’s health consumption. Factors such as the elderly’s chronic diseases and their participation in social interaction influence the effect of depression on the elderly’s health consumption behavior. 

### 3.2. Research Methods

#### 3.2.1. Tobit Model

A comprehensive evaluation index of depression for senior citizens is employed to analyze the CES-D scale. It includes ten questions about the mental health of the respondents. Most studies directly use the overall score of the CES-D as an index to measure the mental health status of respondents. However, the simple summation of scores may be arbitrary and subjective because there may be a correlation among the questions in the scale, and each factor has different representativeness to the depression of the interviewees. Therefore, this paper uses SPSS data processing software to analyze the principal components of the ten items in the CES-D. The scores are redistributed, and weight added to the scores to construct a comprehensive evaluation index of depression for senior citizen respondents. To avoid the influence of different dimensions on each index’s importance, the original data is standardized using the “Z-score” method. The rationality of principal component analysis is verified by testing the correlation between the variables after data standardization. The test results show that there is a specific correlation among the factors. Independent principal component factors can be obtained using principal component analysis to reduce the dimensions and re-assign the original index values.

The Tobit model, in this paper, is used to analyze the influencing factors on the health consumption of senior people. As the explained variable, “health consumption expenditure”, in this empirical study has a distinct data truncation phenomenon at 0 value, the Tobit model is used to regress the full sample data, and family size is used as a tool variable to test the possible endogenous problems of the explanatory variable. The Tobit model compresses the samples whose explanatory variable is 0 into a point and regards their probability distribution as a mixed distribution composed of a discrete point and a continuous distribution, namely the following:

Assuming latent variable

$$Y_{i}^{*}=X_{i}^{\prime }\beta +\varepsilon_{i}\varepsilon_{i}\left|X_{i}\sim N\left(0,{\;\sigma }^{2}\right)Y_{i}^{*}\right|X_{i}\sim N\left(X_{i}^{\prime }\beta ,{\;\sigma }^{2}\right)f\left(Y_{i}^{*}\right)=\frac{1}{\sigma }\phi \left(\frac{Y_{i}^{*}-X_{i}^{\prime }\beta }{\sigma }\right)$$

$$Y_{i}=\{\begin{array}{c} Y_{i}^{*},\mathrm{if}\;’\;Y_{i}^{*}>0\\ 0,\mathrm{if}\;’{\;Y}_{i}^{*}\leq 0 \end{array}$$

$$\begin{array}{c} \begin{array}{c} P\left(Y_{i}>0{|X}_{i}\right)=P\left(Y_{i}^{*}>0{|X}_{i}\right) \end{array}\\ \begin{array}{c} =P\left(X_{i}^{\prime }\beta +\varepsilon_{i}>0{|X}_{i}\right)\\ =1-\Phi \left(\frac{-X_{i}^{\prime }\beta }{\sigma }\right)\\ =\Phi \left(\frac{X_{i}^{\prime }\beta }{\sigma }\right) \end{array} \end{array}$$

$$P(\left(Y_{i}=0{|X}_{i}\right)=1-P\left(Y_{i}>0{|X}_{i}\right)\;=1-\Phi \left(\frac{X_{i}^{\prime }\beta }{\sigma }\right)$$

#### 3.2.2. Two-Step Method

In this research, the Tobit model no longer takes MLE as the core, but expands and improves the Heckman two-step method. The main reason is that the calculation of the Heckman two-step method is relatively simple, the estimation results are consistent, and there is no need to consider the problem of initial value. However, the efficiency of the two-step method is not as good as MLE, and this method requires that the explanatory variables of the two equations cannot be exactly the same. This derivation process of the two-step method proves the estimation properties of the two-step method, as well as the problems needing attention in its application. This interpretation of the method takes into account the application environment of the estimation method described in this paper and the properties of the estimated values. Heckman’s two-step regression method is used to estimate the latent variable equation. Given sufficient exogenous constraints, the instrumental variable (IV) is used to estimate the equation. In addition, the estimator in step 1 is brought into the estimator in step 1, and then the standard ML estimator is used to obtain the uniform estimator of the structural equation.

## 4. Data Sources and Variable Selection 

### 4.1. Data Sources

The data for this paper came from the China Health and Retirement Longitudinal Survey (CHARLS), a large interdisciplinary survey conducted by the National Institute for Development at Peking University and jointly conducted by the China Social Science Survey Center of Peking University and the Youth League Committee of Peking University. The CHARLS is targeted at the families and individuals of middle-aged and elderly people aged 45 or above in China. The questionnaire contains personal and family information on health, consumption, emotion, and other aspects, providing a scientific basis for the analysis of population aging in China. Using the data from the 2015 National baseline surveys conducted by the CHARLS, the sample covered 23,000 respondents from a total of 12,400 households in 150 counties and 450 communities (villages) in 28 provinces (autonomous regions and municipalities directly under the Central Government). Since this paper mainly focuses on the health consumption expenditure of the elderly population, only respondents aged 60 and above are selected as research participants.

### 4.2. Variable Settings and Descriptive Statistics

#### 4.2.1. Health Consumption 

Health consumption expenditure is defined in this study as including the purchase of prescription or non-prescription western medicine, treatment with traditional Chinese herbs or traditional methods, vitamins, supplements, health care equipment, and other items. To ensure the robustness of the regression results, total health consumption expenditure was divided into health consumption expenditure and medical consumption expenditure. Thus, the natural logarithm of the actual amount of three indicators (Total Health Expenditure, Health Care Consumption Expenditure, Medical Consumption Expenditure) is taken, plus one, to overcome the contradiction that when the medical cost is one, the natural logarithm is zero, but when the actual indicated amount is zero, the value is still zero. 

#### 4.2.2. Mental Health

The degree of depression in the elderly was used as the core variable to measure their mental health status, and the comprehensive evaluation score of depression degree in the elderly obtained using a principal component analysis was used as the core explanatory variable (mental) to add into the regression model. The higher the value was, the higher the degree of depression was.

### 4.3. Construction of the Comprehensive Index of Depression using Principal Component Analysis

The CES-D scale includes ten items regarding the mental health of the respondents. They are as follows: “upset by small things,” “it’s hard to concentrate when doing things”, “feeling depressed”, “it feels hard to do anything”, “full of hope for the future”, “being afraid”, “sleeping badly”, “feeling happy”, “feeling lonely”, and “feeling unable to move on in life”. The subjects were divided into the following four levels, according to the length of time the described problem lasted within one week: zero points for less than one day, one point for 1–2 days, two points for 3–4 days, and three points for 5–7 days (reverse-scoring was used for the two questions related to positive emotion). The score of each question was added up after the test, and the higher the total score was, the worse the mental health status could be. 

#### 4.3.1. Data Processing and Feasibility Test

In the feasibility test, SPSS statistical software was used to analyze the CHARLS2015 data. The final research methods (described in the previous Section 3.2) were established using this feasibility test, which was designed to avoid the influence of each dimension on the index’s importance. The original data were standardized using the “Z-score” method. The correlation of the variables was tested after data standardization. The test results show that there is a specific correlation among the factors. To test the feasibility of the principal component analysis, KMO sampling appropriateness factor analysis and Bartlett spherical factor analysis were conducted. The results showed that KMO is 0.879, which is higher than 0.6; Bartlett’s spherical test square value is 45,247.997; the significance is 0.000. Therefore, the data of the CES-D scale is suitable for principal component analysis.

#### 4.3.2. Principal Component Analysis Results

The factors with eigenvalues higher than one were extracted from the ten newly generated independent factors as principal components, and those ten factors were reduced to two principal representative components. The SPSS’s factor analysis results show that the first primary component can explain 38.23% of the total variance; the second principal component can explain 12.19% of the total variance. The two factors can explain 50.42% of the total variance. Considering that the meaning of each factor becomes abstract after changing the coordinates, which is not conducive to interpretation, the maximum variance method is used to rotate the selected factors to obtain a rotated factor load matrix. The factors contained in each principal component from the rotated component matrix were selected according to the principle that the factor load was higher than 0.45. The common characteristics of each factor in each principal component were named. The results shown in Table 1 summarize the depression scale containing the following two principal components: the negative emotional factors and the positive emotional factors, in which the negative emotional factors include eight questions from the original scale, and the positive emotional factors include two questions from the original scale.

Further, the scores of each principal component are calculated according to the principal component score coefficient matrix, namely the following:

$$F_{1}=0.199X_{1}+0.216X_{2}+0.218X_{3}+0.209X_{4}-0.143X_{5}+0.176X_{6}+0.155X_{7}-0.034X_{8}+0.166X_{9}+0.169X_{10}$$

$$F_{2}=-0.024X_{1}-0.116X_{2}-0.016X_{3}-0.037X_{4}+0.675X_{5}-0.050X_{6}-0.037X_{7}+0.542X_{8}+0.044X_{9}+0.033X_{10}$$

SPSS generates two principal component variables according to the standardized principal component score, $F_{1},{\;F}_{2}$. On this basis, the degree of interpretation of the factor to the total variance is taken as the weight, and the scores of the two principal component variables of each sample are added together to construct the comprehensive evaluation index F of depression degree, i.e., $F=38.23\%\times {\;F}_{1}+12.19\%\times {\;F}_{2}$. Since the correlation coefficient of all of the variables in the original data is positive, they have the same explanation direction for the degree of depression. Therefore, the higher the F value is, the higher the degree of depression is (see Table 2).

#### 4.3.3. Cognitive Ability

Cognitive ability may also have an impact on health consumption in the elderly. The higher the cognitive ability is, the more discriminative individuals may be towards false marketing and exaggerated claims on the effects of health products, resulting in lower health expenditures. A higher cognitive ability may also enable the elderly to choose suitable health products and services, also impacting their health consumption expenditures. In this paper, the SIMPLE Mental Intelligence Scale (MMSE) has been incorporated into the CHARLS questionnaire and includes the following four dimensions: orientation, memory, copying ability, and computational ability. The comprehensive “cognition” score was interpreted as the variable, “Cognition”, and added into the regression model. The higher the score is, the more representative it is of the respondents’ cognitive ability. 

#### 4.3.4. Social Interaction

Social interaction has a strong impact on the mental health status of the elderly [23]. Social interaction provides older persons with opportunities to enter and integrate into society, which helps them overcome loneliness and anxiety to improve their mental health. Participation in social activities also has an impact on the health consumption of the elderly. Communication and interaction among older persons can broaden their access to information on health products and services, potentially increasing their health consumption. Based on this view, a “social interaction” score was also interpreted as a variable. It is based on the CHARLS questionnaire data regarding the number of types of social interaction projects that respondents participated in during the past month.

#### 4.3.5. Physiological Health Status

The mental health status of the elderly was assessed using their subjective evaluation of their own health status. In the CHARLS questionnaire, this assessment was taken as an indicator to measure physiological health status, and the virtual variable of self-rated health status (Health_self) was constructed. The self-rated results are given a value of one for “excellent,” “very good,” and “good”, and a value of zero for “average,” “bad”, and “very bad”.

#### 4.3.6. Other Control Variables

Since the degree of depression may be correlated with physiological health, social interaction, cognitive ability, and other variables, this paper examines the specific correlation coefficient values and the degree of significance. The results show that the longer the depression lasted, the fewer the number of social interactions, the lower the cognitive level, and the poorer the physical health. Although the correlation between the degree of depression and other variables was significant, at a 1% significance level, the correlation values were all below 0.25, showing a low correlation. The variable settings and descriptive statistics are shown in Table 3 and Table 4, respectively. Table 5 (following) shows the correlation between depression and these other variables. 

## 5. Empirical Analysis of the Effect of Mental Health Status on Health Consumption 

### 5.1. Baseline Regression

Table 6 shows the regression results of the Tobit model based on the CHARLS2015 national baseline survey’s cross-section data. To ensure the robustness of the empirical results, Model 1, Model 2, and Model 3 show the regression results using the logarithm of the monthly total health consumption expenditure of the elderly, the logarithm of the monthly health consumption expenditure, and the logarithm of the monthly medical consumption expenditure as explained variables, respectively.

The regression results show that depression among the elderly respondents had a positive impact on their monthly total health consumption expenditure, and the result was significant at a 1% level, that is, the higher the depression index was, the higher the total health consumption expenditure was. For each high score of the depression index, the total monthly health consumption expenditure of the elderly increases by 1.61% on average. The results of the sample regression show that the influence of depression on the health care consumption of the elderly is greater than that of medicine consumption. For each point higher in the depression evaluation index, the monthly health care consumption expenditure of the elderly increases by 1.80% on average, while the monthly medical consumption expenditure increases by 1.69% on average.

Participation in social activities has a significant impact on the amount of health consumption expenditure of the elderly. The more social activities the elderly participate in, the greater their health consumption expenditure is. For each additional social activity elderly people participate in per month, their monthly health consumption expenditure will increase by 0.29%, 1.06% and 0.16%, respectively. The regression result is significant at the 1% level.

Physiological health has a significant impact on the health consumption of the elderly. It can be found from the regression results that the monthly total health consumption expenditure of the elderly with a good self-rated health status is 1.98% lower than that of the elderly with a poor self-rated health status; the monthly health consumption expenditure is 2.25% lower, and the monthly medical consumption expenditure is 2.11% lower, that is, the elderly with a worse physical health status tend to consume more health products. The regression results were significant at the 1% level.

It can also be found from the regression results that, in addition to the above factors, other control variables have a significant impact on the health consumption expenditure of the elderly. For example, the average monthly health consumption expenditure of women is significantly higher than that of men, which is consistent with the conclusion that the depression rate is higher in women than it is in men [24], and is consistent with women’s low evaluation of their health status (see Table 6). In addition, respondents living in urban areas, with retirement plans, and medical insurance spend significantly more on health products.

### 5.2. Endogeneity Test

The Tobit two-step method for estimation was used to test the endogeneity of the model. Happiness has an important impact on the mental health of the elderly. Those who feel happy in life tend to be less depressed and for shorter periods of time. Happiness, however, may not directly affect levels of health consumption. Therefore, this paper uses the questionnaire results of respondents that are happy to construct the “Happiness variable”, described as “Satisfaction”. The regression results are shown in Table 7.

All of the models controlled for depression, cognitive level, social activity, health status, gender, age, age squared, education level, retirement, social security, personal monthly income, and resident variables. The IV Tobit two-step regression passed the Wald test and rejected the exogenous hypothesis; the coefficient of “Satisfaction”, the instrumental variable in the first step regression, was −0.41 and significant at the 1% level, and the F value of the whole equation was 92.64; therefore, there was no weak instrumental variable problem. It was found that the IV Tobit regression results for the depression degree coefficient values were significantly positive, indicating that the degree of depression in elderly health spending is significant. There were no significant differences in the rest of the regression coefficients of each variable between the two regression results, suggesting that the regression results are reliable.

### 5.3. The Regulating Effects of Chronic Diseases 

The mental health status of the elderly may be affected by their physiological health status. The results of the regression analysis show that health consumption can relieve depression, but those who suffer from a chronic disease are more cautious in selecting and purchasing health products. Their health consumption expenditure is more determined by doctors’ orders than their own emotional state. They may choose to relieve the pain of depression in ways other than health consumption. To test whether chronic disease has a regulating effect on depression scores, a baseline regression was added. The regression results show that chronic disease can significantly reduce the effect of depression on the elderly’s health consumption. The regulating effect of chronic disease on the depression of the elderly in medical expenditure is particularly prominent, as shown in Table 8.

### 5.4. The Need to Socialize

Social interaction may also influence the extent to which mental health status contributes to health consumption. Activities such as chatting with friends, playing cards, and dancing might alleviate depression, and respondents who participate in fewer types of social activities may spend more on health consumption due to long-term depression. The results of the regression analysis show that the correlation between social activity and depression is significantly negative. People who participate in social activities will spend more on health consumption. The results are shown in Table 9. 

### 5.5. Urban–Rural Differences in Health Consumption 

There is a gap between the per capita medical and health consumption expenditure of rural citizens and that of urban citizens. This gap is a result of the differences in income and social security. The disparity among elderly urban and rural residents was examined in an additional regression analysis. The results show that there is a significant difference in urban and rural health consumption. An urban residency has a stronger effect on health consumption. There is also a gap in cognitive ability and its impact on health consumption. Even though urban residents show a higher cognitive ability, it was the respondents living in rural areas that demonstrated a significant correlation between health consumption expenditure—particularly medical expenditure—and cognitive ability. A wider array and the greater availability of health products may confront the urban elderly with a stronger risk awareness, motivating them to use medication from hospitals according to the prescription of professional doctors. The results of this analysis are shown in Table 10.

## 6. Conclusions

In this paper, the CHARLS2015 national baseline survey data were used to calculate the comprehensive evaluation index of the degree of depression in the elderly in China through principal component analysis. The Tobit model was employed to study the impact of mental health status on the health consumption of the elderly in China, and the mechanism of action was analyzed. The study found that the degree of depression had a significant impact on the health consumption of the elderly.

### 6.1. The Overall Effect of Mental Health Status on Health Consumption 

Depression has a significant positive effect on the health consumption of the elderly. Long-term depression will encourage the elderly to seek effective ways to relieve the pain caused by negative emotions and increasing their health consumption is an important way for the elderly to alleviate depression. The cognitive level and education level have a significant positive effect on the health consumption of the elderly. Elderly people with a strong cognitive ability are better at choosing suitable health products for consumption. In contrast, for the elderly with a weak cognitive ability, it is difficult to judge their need for health products and effectively identify and purchase health products, resulting in a lower health consumption expenditure. The more the elderly participate in social activities, the higher their health consumption expenditure will be. Social activities provide a channel for the elderly to understand consumption information, and interaction and communication between the elderly may also improve their enthusiasm for health consumption.

### 6.2. The Moderating Effects of Chronic Diseases, Social Activities, and Health Consumption 

Elderly people who have experienced negative emotions for a long time seem to increase their health consumption in order to alleviate them. Chronic disease and social activities are variables that act on the effects of mental health on health consumption. The results show that chronic disease significantly reduces the effects of depression on health consumption. If older people suffer from multiple chronic diseases, their health spending is determined more by medical advice than by their emotional state. Those who participate in more social activities will consume less health products because social activity works as a mitigating factor against depression. 

### 6.3. Urban–Rural Differences Impact the Mental Health Status and Health Consumption 

There are differences between rural and urban residency regarding mental health and health consumption. A higher income and better social security result in a bigger budget for health consumption and for mitigating the effects of depression. In urban areas, the demand for health products is met with more variety and better provision of such goods and services, compared to rural areas. Elderly residents in rural areas may suffer more from depression and will also tend to increase their spending on health consumption, but for different reasons. 

### 6.4. Suggestions

Mental health issues among the elderly are crucial to public health and national security. Health consumption is a way that the elderly people often choose to relieve depression, and the long-term depression of the elderly has become one of the main reasons for increasing health consumption. On the one hand, this phenomenon may lead to excessive consumption of the elderly, and irrational and blind consumption may aggravate the mental health problems of the elderly. On the other hand, a good health consumer market plays an important role in improving the mental health of the elderly. More research on the effects of an aging population and its health are necessary, but the findings in this study can contribute to the better management of health consumption among the elderly. Key findings include the following suggestions: pay attention to the mental health problems of the elderly; attach importance to the improvement of the cognitive ability of the elderly; promote the development of the health consumer market; and strengthen the construction of a health information system for the elderly. These suggestions are discussed in the following sections. 

#### 6.4.1. Pay Attention to the Mental Health Problems of the Elderly 

Effective leadership and management of mental health work for the elderly can be strengthened by providing public venues for social activities. This includes the platforms for social interaction that broaden their channels for alleviating negative emotions. Elderly individuals can be encouraged to join universities for the elderly and other interactive groups to enrich their lives while improving their ability to deal with psychological problems and increase their cognitive ability. Policymakers should attach importance to social support for the elderly; formulate scientific and reasonable community mental health service policies; allocate professionals in communities, medical institutions, and other places to provide psychological counseling and other services for the elderly; encourage cooperation between communities and hospitals; and provide timely professional guidance and scientific treatment programs for the mental health of the elderly. It has also been observed that household characteristics strongly affect health consumption. Leaders in healthcare should pay special attention to strengthening the existing family support networks [25]. 

#### 6.4.2. Attach Importance to the Improvement of the Cognitive Ability of the Elderly

Cognitive ability has an important influence on the consumer demand for health products. Those with a higher cognitive ability tend to be more selective in using judgment to choose and buy health products. They seek better quality products and are less inclined to be misled by bad-intentioned sales and marketing gimmicks. Policymakers should focus on providing improved comprehensive and integrated mental health and social care services for the elderly in a community-based environment. The government should formulate relevant social security policies to provide the elderly with necessary health knowledge and economic support and encourage grass-roots organizations to offer artistic and sports activities for the elderly to enrich their social life and help them consolidate and improve their cognitive abilities in the process of interacting with others. Decision makers should work to cultivate the attitudes of the elderly to cope with aging actively; organize medical experts to popularize health knowledge through media and offline publicity activities; provide financial support for the regular physical examination of the elderly; and guide the elderly to have a scientific and accurate understanding of their health status. Opening lecture halls for the aged, developing community education for the aged, and lifelong learning behavior contribute to physical health and mental well-being [26].

#### 6.4.3. Promote the Development of the Health Consumer Market 

The quality review system of health products and the supervision and management system of the health consumption market should be improved to prevent health product businesses from taking advantage of the psychological characteristics of the elderly through exaggerated propaganda, false marketing, and over-pricing, and regulate the health consumption market from the supply side. The medical care consultation service system should give play to the power of the government, society, community, and family to help the elderly improve their health awareness by establishing correct concepts of health consumption and guide the healthy consumption market to develop from the demand side. The elderly population’s demand for health products should be improved to realize the sustainable growth of the health consumption market and create a virtuous cycle environment for the elderly’s medical and health consumption, while at the same time, formulate preferential policies to guide the distribution of health product brands in rural areas, expand the rural health consumption market, and provide a greater choice space for the rural elderly’s health consumption.

#### 6.4.4. Limitations and Future Research: Strengthening Health Information Systems 

The availability of data and health index designs limit public understanding on the impact of mental health status on the health consumption of the elderly in China. To strengthen the construction of health information system for the elderly population, the Chinese government should increase the special fund investment in the construction of citizens’ health records; accelerate the construction and improvement of the evaluation index system of citizens’ physical and mental health; and ensure the rationality of the micro questionnaire design, the effectiveness of household visits, and the consistency of statistical caliber. Statistical departments should strengthen the monitoring and sharing of data with the health sector, improve the health records of the elderly, ensure the continuity and reliability of data, and increase the frequency of updating health data. Universities and research institutions should make full use of the consumption and health data resources of the elderly; set up research topics on the health consumption of the elderly; mine the academic research value of the data; conduct international exchanges; and build a platform for sharing policies, experiences, and best practices on healthy aging [27]. Better information and better communication between researchers and policymakers can lead to a better quality of life for China’s aging population. 

Future studies should focus on examining how health care consumption is related to other expenditures that can be of a very different nature, including alcohol and drug abuse [28] or rising complications due to the COVID-19 pandemic [29]. There may also be significant differences in health consumption across genders [30], especially concerning issues such as affective symptoms, insomnia, and burnout or other maladaptive coping strategies that may or may not be culture specific. Future research also needs to focus on comparative studies that investigate the problems of prescription drug use (particularly antidepressants) in other countries and other endemic health issues [31]. In addition, other countries have also conducted national health surveys [32] and important findings might be found in future comparative studies on an international level. This direction in research might also extend the readership of China-based research to a wider readership. 

## Figures and Tables

**Table 1:** Results of the principal component analysis.

Factor/Variable Name	Factor Load	The Eigenvalue	Explanatory Variance
Factor 1: Negative emotional factors		3.82	38.23%
Feel in low spirits	0.772		
It is hard to do anything	0.725		
Worry about little things	0.698		
It is hard to concentrate	0.691		
I feel like I cannot go on with my life	0.632		
Feel lonely	0.628		
Feel afraid	0.597		
Poor sleep	0.531		
Factor 2: Positive emotional factors		1.22	12.19%
Feel happy	0.758		
Full of hope for the future	0.871		

**Table 2:** Principal component score coefficient matrix.

Factors	Principal Component Score Coefficient	
	Principal Component 1	Principal Component 2
Worry about little things	0.199	0.024
It is hard to concentrate	0.216	0.116
Feel in low spirits	0.218	0.016
It is hard to do anything	0.209	0.037
Full of hope for the future	0.143	0.675
Feel afraid	0.176	0.050
Poor sleep	0.155	0.037
Feel happy	0.034	0.542
Feel lonely	0.166	0.044
I feel like I cannot go on with my life	0.169	0.033

**Table 3:** Variable Settings.

Classification	Variable Name	Variable Declaration
Healthy Consumption	Logarithm of Total Health Expenditure (Lnexp)	The logarithm of the total amount of health expenditure of the elderly respondents in the last month, and the value of 0 is 0. Health consumption items include elderly people’s independent purchase of prescription or non-prescription western medicine, treatment with traditional Chinese herbs or traditional methods, vitamins, supplements, and health care equipment.
	The Logarithm of Health Care Consumption Expenditure (Lnhexp)	The logarithm of the total amount of health care expenditure of the elderly respondents in the last month, and the value of 0 is 0. The health consumption item includes the elderly’s own purchase of health products and health equipment.
	The Logarithm of Medical Consumption Expenditure (Lndexp)	The logarithm of the total amount of medical expenditure of the elderly respondents in the last month. If the amount of expenditure is 0, the value is 0. Medical consumption items include the elderly’s independent purchase of prescription or non-prescription western medicine, traditional Chinese herbal medicine, and traditional treatment.
Mental Health	Degree of Depression (Mental)	Comprehensive evaluation score concluded of the depression degree of the elderly respondents, which was evaluated using Principal component analysis.
Cognition and Social Interaction	Cognition.	Comprehensive evaluation score concluded of the cognitive ability of the elderly respondents, which was evaluated using Principal component analysis.
	Social Interaction (Social_Num)	The types of social activities the elderly respondents had participated in in the past month.
Physical Health	Self-Rated Health Status (Health_Self)	The self-rated results are given a value of 1 for “excellent,” “very good”, and “good”, and a value of 0 for “average”, “bad”, and “very bad.”
	Chronic Diseases (Diseases)	The number of types of chronic diseases of the elderly interviewed/the total number of chronic diseases.
Other Control Variables	Gender (Male)	Men are assigned a value of 1 and women are assigned a value of 0.
	Age (Age)	The actual age of the elderly respondents.
	Age Squared (Age2)	The square of the respondent’s real age.
	Education Level (Edu)	The number of years of schooling of the elderly respondents.
	Emeritus (Retire)	The procedure for retirement (including resignation) is 1, otherwise, it is 0.
	Social Security	The number of elderly respondents covered by health insurance.
	Personal Monthly Income (Lnincome)	After deducting income tax, insurance, housing accumulation fund, and other expenses, the sum of the average monthly salary and the average monthly transfer income received by the elderly respondents is taken as the logarithm, and the value of 0 is 0.
	Local (Address)	Towns are assigned a value of 1, and villages are assigned a value of 0

**Table 4:** Descriptive statistical results based on national baseline survey data in 2015.

Classification	Variable Name	Sample Size	Mean	Standard Deviation	Minimum Value	Maximum
Healthy Consumption	Exp	8573	1980.666	3883.275	0	101,091.7
	Hexp	8573	46.713	540.321	0	20,000
	Dexp	8573	131.074	449.121	0	20,000
Mental Health	Getting	18,089	0.00	0.401	0.43	1.43
Cognition and Social Interaction	Cognition	12,887	0.00	0.468	1.53	1.55
	Social_num	8573	0.83	1.065	0	9
Physical Health	Health_self	8571	0.21	0.408	0	1
	Diseases,	18,089	0.09	0.105	0	0.71
Other Control Variables	Male	8572	0.51	0.500	0	1
	Age	8071	67.80	6.347	60	102
	Age2	8071	4636.55	898.715	3600	10,404
	Edu	8572	1.63	2.386	0	56
	Retire	8048	0.17	0.374	0	1
	Insurance	8573	0.98	0.459	0	8
	Lnincome	8516	1.40	2.504	0	9.47
	The Address	8550	0.30	0.456	0	1

**Table 5:** Correlation between depression and other explanatory variables.

	Social Interaction	Cognitive Ability	Chronic Diseases	Self-Assessment of Health Status
The Degree of Depression	0.110 *** (8573)	0.210 *** (12,333)	0.218 *** (18,089)	0.228 *** (8571)

Note: Correlation coefficient is Pearson’s correlation coefficient; in brackets are the number of pairwise related samples *** significant at the 1% significance level.

**Table 6:** Regression results of the Tobit model.

	(1)	(2)	(3)
	Lnexp	Lnhexp	Lndexp
Mental	1.612 *** (0.180)	1.804 *** (0.598)	1.685 *** (0.185)
Cognition	0.485 *** (0.158)	1.536 *** (0.528)	0.347 ** (0.164)
Social_num	0.294 *** (0.0582)	1.057 *** (0.175)	0.162 *** (0.0608)
Health_self	1.976 *** (0.171)	2.245 *** (0.596)	2.106 *** (0.179)
Male	0.314 ** (0.137)	2.252 *** (0.453)	0.0656 (0.142)
Age	0.591 *** (0.209)	1.436 ** (0.708)	0.469 ** (0.217)
Age^2^	0.00397 *** (0.00149)	0.00905 * (0.00504)	0.00323 ** (0.00155)
Edu	0.0512 ** (0.0249)	0.0312 (0.0792)	0.0560 ** (0.0258)
Retire	0.741 *** (0.183)	2.736 *** (0.573)	0.467 ** (0.190)
Insurance	0.538 *** (0.141)	1.215 *** (0.422)	0.467 *** (0.147)

Note: ***, **, * at the level of 1%, 5%, and 10%, respectively; standard error in brackets. The table shows the coefficients corresponding to each variable.

**Table 7:** Results of Tobit two-step estimation.

	IV Tobit Two-Step Method		Tobit
	Step1	Step2	
	Getting	Lnexp	Lnexp
Satisfaction	0.411 *** (0.0276)		
Mental		1.455 ** (0.652)	1.612 *** (0.180)
Cognition	0.132 *** (0.0118)	0.458 ** (0.182)	0.485 *** (0.158)
Social_Num	0.00670 * (0.00399)	0.290 *** (0.0584)	0.294 *** (0.0582)
Health_Self	0.170 *** (0.0105)	2.003 *** (0.208)	1.976 *** (0.171)
Other Control Variables	control	control	control
Constant Term	1.359 *** (0.518)	21.31 *** (7.330)	21.06 *** (7.282)
Sample Size	4666	4666	4674

Note: ***, **, * at the level of 1%, 5%, and 10%, respectively; standard error in brackets. The first step regression results of the two-step IV Tobit method report the robust standard error of the ordinary least square regression, and the second step regression results and Tobit regression results report the standard error in brackets. Full regression results are available from the author due to space limitations. The table shows the coefficients corresponding to each variable.

**Table 8:** Regulating effect of chronic disease on mental health and health consumption.

	(1)	(2)	(3)
	Lnexp	Lnhexp	Lndexp
Mental	2.239 *** (0.259)	2.284 ** (0.889)	2.322 *** (0.268)
Diseases	8.535 *** (0.583)	7.404 *** (1.843)	8.844 *** (0.605)
Mental Diseases	4.802 *** (1.300)	2.851 (4.147)	4.830 *** (1.341)
Control Variables	control	control	control
Constant Term	20.75 *** (7.227)	67.52 *** (24.80)	16.43 ** (7.488)
Number of Samples	4674	4674	4672
Pseudo R^2^	0.026	0.046	0.023

Note: ***, **, * at the level of 1%, 5%, and 10%, respectively; standard error in brackets.

**Table 9:** The effect of social activity participation on health consumption.

	(1)	(2)	(3)
	Lnexp	Lnhexp	Lndexp
Mental	1.803 *** (0.221)	2.326 *** (0.742)	1.714 *** (0.228)
Social num	0.273 *** (0.0599)	1.000 *** (0.181)	0.159 ** (0.0623)
Mental Social num	0.246 * (0.165)	0.573 * (0.487)	0.0375 (0.172)
Control variables	control	control	control
Constant term	21.03 *** (7.280)	67.92 *** (24.76)	16.53 ** (7.540)
Number of samples	4674	4674	4672
The pseudo R^2^	0.022	0.046	0.019

Note: ***, **, * at the level of 1%, 5%, and 10%, respectively; standard error in brackets.

**Table 10:** Urban-rural differences in health consumption.

	City sample			Rural samples		
	Lnexp	Lnhexp	Lndexp	Lnexp	Lnhexp	Lndexp
Mental	1.739 *** (0.338)	2.041 ** (0.970)	1.746 *** (0.353)	1.549 *** (0.211)	1.758 ** (0.767)	1.643 *** (0.216)
Cognition	0.187 (0.286)	2.417 *** (0.829)	0.0298 (0.301)	0.630 *** (0.190)	0.927 (0.690)	0.537 *** (0.195)
Social_num	0.289 *** (0.0850)	1.076 *** (0.226)	0.0907 (0.0902)	0.319 *** (0.0811)	0.984 *** (0.277)	0.256 *** (0.0835)
Health_self	1.975 *** (0.280)	2.562 *** (0.847)	2.207 *** (0.298)	1.982 *** (0.217)	1.840 ** (0.835)	2.059 *** (0.224)
Male	0.280 (0.230)	3.031 *** (0.671)	0.213 (0.242)	0.365 ** (0.171)	1.597 ** (0.621)	0.255 (0.176)
Age	0.570 * (0.345)	1.391 (1.024)	0.435 (0.362)	0.619 ** (0.265)	1.519 (0.987)	0.500 * (0.272)
Age^2^	0.00374 (0.00245)	0.00859 (0.00728)	0.00294 (0.00257)	0.00423 ** (0.00190)	0.00968 (0.00704)	0.00349 * (0.00195)
Edu	0.0491 (0.0357)	0.0353 (0.100)	0.0690 * (0.0375)	0.0518 (0.0351)	0.0311 (0.128)	0.0456 (0.0361)
Retire	0.631 ** (0.248)	2.338 *** (0.733)	0.257 (0.260)	1.040 *** (0.286)	3.211 *** (0.925)	0.953 *** (0.294)
Insurance	0.484 ** (0.215)	1.038 * (0.609)	0.480 ** (0.226)	0.536 *** (0.189)	1.435 ** (0.594)	0.389 ** (0.197)
Lnincome	0.0343 (0.0420)	0.126 (0.121)	0.0182 (0.0441)	0.00443 (0.0319)	0.0209 (0.118)	0.0114 (0.0328)
Constant term	20.15 * (12.05)	65.10 * (35.82)	15.12 (12.64)	21.72 ** (9.199)	71.72 ** (34.50)	17.25 * (9.435)
Number of samples	1639	1639	1638	3035	3035	3034
The pseudo R^2^	0.019	0.043	0.018	0.021	0.027	0.020

Note: ***, **, * at the level of 1%, 5%, and 10%, respectively; standard error in brackets.
